# Inflammaging links Life's Essential 8 to white-matter brain ageing

**DOI:** 10.1016/j.ebiom.2025.105908

**Published:** 2025-09-03

**Authors:** Franco Grimolizzi

**Affiliations:** Division of Molecular Nutrition, Department of Nutrition, Institute of Basic Medical Sciences, University of Oslo, Oslo, Norway

Dear Editor,

Feng et al. show that every 10-point rise in Life's Essential 8 (LE8) keeps white matter nearly four months “younger,” a finding clinicians can act on immediately.^1^ One mechanistic thread, however, could be explored further: inflammaging—the chronic, smouldering inflammation that accompanies biological ageing and gradually erodes white-matter integrity.^2^^,^^3^

Several of the authors’ own results already hint at this axis. The muted LE8 benefit in APOE4 carriers, especially midlife women, mirrors sex- and genotype-specific inflammatory responses to metabolic stress.^4^ The age-stratified curves also hint at an inflammatory shift after the age of 60. Yet without standard serum inflammatory markers, the link between lifestyle and brain ageing remains largely inferential.

Including a modest panel—C-reactive protein, interleukin-6, and glycoprotein acetyls—at the next UK Biobank imaging wave could test a simple pathway: *higher LE8 → lower inflammaging → younger white matter*. Demonstrating this would (i) help identify people whose brains age “hotter” than their arteries, (ii) support giving greater weight to anti-inflammatory habits such as good sleep hygiene and plant-forward diets within LE8, and (iii) open the door to pragmatic trials pairing lifestyle coaching with safe vascular anti-inflammatories^5^ (e.g., low-dose colchicine).

Framing future analyses around inflammaging would strengthen the biological plausibility of the LE8–white-matter association and clarify which individuals might benefit most.

## Contributors

FG conceived the idea, conducted the literature review, and drafted the manuscript. FG reviewed and approved the final version of the manuscript.

## Declaration of interests

The author declares no competing interests.
